# Integrating Prevention of Mother-to-Child HIV Transmission Programs to Improve Uptake: A Systematic Review

**DOI:** 10.1371/journal.pone.0035268

**Published:** 2012-04-27

**Authors:** Lorainne Tudor Car, Michelle H. M. M. T. Van Velthoven, Serena Brusamento, Hoda Elmoniry, Josip Car, Azeem Majeed, Peter Tugwell, Vivian Welch, Ana Marusic, Rifat Atun

**Affiliations:** 1 Department of Primary Care and Public Health, School of Public Health, Imperial College London, London, United Kingdom; 2 Centre for Global Health, University of Ottawa, Ottawa, Canada; 3 Department of Research in Biomedicine and Health, University of Split School of Medicine, Split, Croatia; 4 Imperial College Business School, Imperial College London, London, United Kingdom; University of Cape Town, South Africa

## Abstract

**Background:**

We performed a systematic review to assess the effect of integrated perinatal prevention of mother-to-child transmission of HIV interventions compared to non- or partially integrated services on the uptake in low- and middle-income countries.

**Methods:**

We searched for experimental, quasi-experimental and controlled observational studies in any language from 21 databases and grey literature sources.

**Results:**

Out of 28 654 citations retrieved, five studies met our inclusion criteria. A cluster randomized controlled trial reported higher probability of nevirapine uptake at the labor wards implementing HIV testing and structured nevirapine adherence assessment (RRR 1.37, bootstrapped 95% CI, 1.04–1.77). A stepped wedge design study showed marked improvement in antiretroviral therapy (ART) enrolment (44.4% versus 25.3%, p<0.001) and initiation (32.9% versus 14.4%, p<0.001) in integrated care, but the median gestational age of ART initiation (27.1 versus 27.7 weeks, p = 0.4), ART duration (10.8 versus 10.0 weeks, p = 0.3) or 90 days ART retention (87.8% versus 91.3%, p = 0.3) did not differ significantly. A cohort study reported no significant difference either in the ART coverage (55% versus 48% versus 47%, p = 0.29) or eight weeks of ART duration before the delivery (50% versus 42% versus 52%; p = 0.96) between integrated, proximal and distal partially integrated care. Two before and after studies assessed the impact of integration on HIV testing uptake in antenatal care. The first study reported that significantly more women received information on PMTCT (92% versus 77%, p<0.001), were tested (76% versus 62%, p<0.001) and learned their HIV status (66% versus 55%, p<0.001) after integration. The second study also reported significant increase in HIV testing uptake after integration (98.8% versus 52.6%, p<0.001).

**Conclusion:**

Limited, non-generalizable evidence supports the effectiveness of integrated PMTCT programs. More research measuring coverage and other relevant outcomes is urgently needed to inform the design of services delivering PMTCT programs.

## Introduction

At the United Nations General Assembly Special Session (UNGASS) in 2001, a goal was set to reduce by 2010 the proportion of HIV infected infants by 50%. Achieving this target would require that an estimated 80% of pregnant women and their children need to receive essential HIV prevention, treatment and care [1].

Women represent slightly more than 50% of HIV infected population [2]. Each day, an estimated 1000 children under the age of 15 acquire HIV infection [2]. More than 90% of HIV infections in children under the age of 15 are due to mother-to-child transmission (MTCT) of HIV and more than 90% of the mother to child transmission occurs in sub-Saharan Africa [2]. Transmission of HIV from mother-to-child can be reduced from 15–40% to 1% through effective prevention of mother-to-child transmission of HIV (PMTCT) programs [3].

When PMTCT programs were initially introduced in low- and middle-income countries, they were stand-alone programs, with gradual integration into maternal and newborn healthcare services [4], [5]. The rationale for integrating PMTCT interventions with other healthcare services is to improve access for women and children and increase the quality of care through better and synergistic use of human and financial resources. Furthermore, implementing a PMTCT program as a part of routine healthcare helps reduce the stigma experienced by HIV infected people [6]–[8]. However, many women in low- and middle-income countries lack access to maternal and child healthcare services [9]. Furthermore, even pregnant women attending maternal and child healthcare services are often not reached by all requisite interventions of the PMTCT programs integrated with these services. Additionally, integration could overburden already weak healthcare services in resource-limited countries. Lack of resources, leadership and monitoring could have a negative impact on the implementation and sustainability of the integrated services. Integration could also lead to increased stigmatization towards the healthcare services providing PMTCT interventions and clients attending those services [10], [11].

The existing integrated PMTCT programs experience attrition at each step of the program delivery: from the first contact, through counseling, testing, collecting results, receiving antiretroviral therapy (ART), infant treatment and feeding recommendation and postnatal follow-up; thereby reducing program effectiveness [12]–[14]. Based on data from a multi-country PMTCT program implemented by the Elizabeth Glaser Pediatric AIDS Foundation in 2006, of 100 pregnant women that attend antenatal clinic, 92 are counseled, 77 are tested for HIV and 69 receive test results. Of eight women identified as HIV positive, six receive ARV prophylaxis. Of infants born to these eight HIV positive women identified, only four will receive prophylaxis [15]. In 2009, only an estimated 26% of pregnant women in low- and middle-income countries were tested for HIV and an estimated 53% [40%–79%] of them received antiretroviral medication to prevent the mother-to-child transmission of HIV [12]. In 11 of the 25 countries with the largest numbers of women needing prophylaxis with antiretroviral medicines to reduce mother-to-child transmission, coverage levels were below 50% [12].

Integration of PMTCT with other healthcare services is a crucial component of the strategy to scale up PMTCT programs [12], [16]–[18]. However, there is no universally accepted definition of integrated care [19]–[23]. “Integration” is used to describe a range of organizational arrangements with variable nature and intensity [24]. Therefore, any analysis and comparison of integrated healthcare is methodologically challenging. Research on integration of healthcare services into health systems to improve health outcomes has been mostly focused on comparison between integrated or horizontal versus non-integrated or vertical programs. However, it has also included additional concepts such as diagonal or oblique programs for those programs which are neither fully integrated nor completely vertical, acknowledging that the extent of integration of services can vary greatly. Furthermore, it has been accepted that integration can happen at various levels of the health system, i.e. local, district, regional or national and can include different health system functions such as governance, financing, planning, service delivery, monitoring and evaluation, and demand generation [24]. In this review, we defined the integration of PMTCT interventions as joining of service delivery of PMTCT programs with other healthcare services at a single point of access [25]. We defined partially integrated PMTCT programs as those that refer women to a separate facility for PMTCT interventions and those that integrate a segment of the PMTCT program compared to the intervention arm. By non-integrated PMTCT programs, we considered separate, detached, stand-alone PMTCT programs without any links to or referrals from other healthcare services.

Our preliminary searches found a systematic review on integration of sexual and reproductive health services with HIV services produced by the WHO in 2009 [25]–[27]. The review, framed around the ‘four prongs’ of PMTCT defined by WHO, focused on integration of the first, the second and the fourth prong of PMTCT strategies while the third prong was - very surprisingly - excluded if the interventions were not linked to other areas of sexual and reproductive health.

This left a substantial gap in evidence provided by this report. In addition to this systematic review, we found several non-systematic literature overviews [28]–[31]. Given the importance of the question and weak evidence base, there is an urgent need to systematically evaluate effectiveness of integrated compared to non- or partially integrated PMTCT programs on the uptake of PMTCT interventions.

## Methods

### Search Strategy

We performed a comprehensive search to identify peer-reviewed academic and grey literature published between January 1990 (i.e. the year first PMTCT programs were established) and August 2010 [32]. We used a sensitive search strategy developed in collaboration with the Cochrane HIV/AIDS Review Group. We used the search string in Table 1 for Medline (PubMed). Details of the search strategies for other databases are available from the authors.

**Table 1:** Search strategy MEDLINE (PubMed).

#1 Search HIV Infections[MeSH] OR HIV[MeSH] OR hiv[tw] OR hiv-1*[tw] OR hiv-2*[tw] OR hiv1[tw] OR hiv2[tw] OR hiv infect*[tw] OR human immunodeficiency virus[tw] OR human immunedeficiency virus[tw] OR human immuno-deficiency virus[tw] OR human immune-deficiency virus[tw] OR ((human immun*) AND (deficiency virus[tw])) OR acquired immunodeficiency syndrome[tw] OR acquired immunedeficiency syndrome[tw] OR acquired immuno-deficiency syndrome[tw] OR acquired immune-deficiency syndrome[tw] OR ((acquired immun*) AND (deficiency syndrome[tw])) OR “sexually transmitted diseases, viral:noexp”[MH]
#2 Search mother-to-child[tiab] OR MTCT[tiab] OR mother-to-infant[tiab] OR adult-to-child[tiab] OR maternal-to-child[tiab] OR vertical transmission[tiab] OR perinatal transmission[tiab] OR postnatal transmission[tiab] OR post natal transmission[tiab] OR maternal-infant transmission[tiab] OR PMTCT[tiab] OR infectious disease transmission, vertical/prevention and control[mh]
#3 Search #1 AND #2
#4 Search (#1 AND #2) NOT (animals[mh] NOT humans[mh])
#5 Search (#1 AND #2) NOT (animals[mh] NOT humans[mh]) Limits: Publication Date from 1990/01/01 to 2010/07/26

The search strategy did not entail search terms related to integrated care because in some studies “integration” could have been omitted or labeled differently. Instead, we examined service delivery in each study to decide if it would meet our definition of healthcare service integration.

We searched MEDLINE, EMBASE, the Cochrane Library, the WHO Global Health Library, CAB abstracts, CINAHL, POPLINE, PsycINFO, Sociological abstracts, ERIC and U.S. National Library of Medicine Gateway System, the WHO International the Clinical Trials Registry and Controlled Clinical Trials. We also searched several grey literature sources: AEGIS, Google Scholar, New York Academy of Medicine Grey Literature, Open SIGLE, British Library Catalogue and ProQuest Dissertation & Theses Database. Google Scholar search resulted in a large number of hits of which we scanned the first 500. Two reviewers independently performed the searches. We screened reference lists of included studies and relevant systematic reviews for additional studies. We contacted authors of relevant conference proceedings for additional information.

### Eligibility Criteria

According to WHO, PMTCT consists of four prongs: 1) primary prevention of HIV infection in women; 2) prevention of unintended pregnancy among HIV-positive women; 3) reduction of transmission from HIV infected pregnant and lactating women to their children during the perinatal period, i.e. pregnancy, birth and infant feeding and care and 4) support of women, infants, and families infected and affected by HIV/AIDS.

Our review assessed the effect of integration of the third prong of PMTCT measures i.e. perinatal PMTCT interventions aimed at reducing MTCT of HIV) These consist of HIV testing of pregnant women, reduction of viral load with antiretroviral therapy (ART) or prophylaxis, safe delivery practices, infant feeding counseling, infant antiretroviral (ARV) prophylaxis and HIV testing of infants. Safe delivery practices are defined as elective caesarean section or vaginal delivery with minimal use of invasive procedures.

We considered eligible studies focused on integration of any, including more than one, of the perinatal PMTCT interventions with other healthcare services.

Eligible controls were usual care, consisting of non-integrated or partially integrated healthcare services.

We included all study designs with a control or comparison in order to present all existing evidence on the effectiveness of integration of PMTCT programs. We included experimental, quasi-experimental and controlled observational studies. While in observational studies it is never possible to rule out unrecognized bias with confidence, they help us understand the issues studied [33]. Studies had to focus on pregnant women, women in labor and postpartum and their infants, with unknown or positive HIV status, from low- and middle-income countries as defined by the World Bank [34], with the proportion of women and children receiving individual PMTCT interventions as a primary outcome. In addition, we aimed to collect other relevant outcomes such as the cost-effectiveness and impact of integrated care on human resources, quality of care, stigma and attendance of services integrated with PMTCT. We included studies in any language. Our review was informed by a Cochrane review we did in parallel with the Cochrane HIV/AIDS Group [35]. There were several differences between this and the review registered with the Cochrane Collaboration: type of the included studies, quality assessment tools, analysis of the data etc. The protocol peer-review performed by the Cochrane Collaboration helped us with this review. In particular in relation to the study which met Cochrane criteria (one study) [36], [37].

### Screening Process, Data Extraction and Synthesis

Two review authors independently performed screening of the citations and data extraction. Disagreements between authors were resolved by discussion, consensus and consultation with a third review author. Citations were downloaded into bibliographic management software (EndNote X4). We screened the titles and abstracts and then full-texts of the studies for eligibility. We extracted data from the included studies using a standardized data extraction sheet, which summarized key information from the relevant studies, such as administrative data, methodology, information on participants, interventions, outcomes, comparison and other notes. Statistical pooling of results was not feasible due to heterogeneity of reported interventions, outcomes and study designs. Therefore, we present the findings narratively.

### Quality Assessment

Two review authors separately performed the assessment of the quality of the evidence. Any differences of opinion were resolved by discussion and in consultation with the third review author.

We used a three-point scale examining the suitability of the study design. This was adapted from the criteria used for the Community Guide of the US Task Force on Community Preventive Services [38].

For randomized controlled trials, controlled clinical trials, cluster randomized controlled trials, controlled before and after studies and interrupted time series, we used the risk of bias tool developed by the Cochrane Collaboration [39]–[41]. Each criterion had to be rated as either yes (low risk of bias), unclear (uncertain risk of bias), or no (high risk of bias).

We evaluated other study designs by a six-item checklist of criteria developed for the Effective Public Health Practice Project in Hamilton, Ontario [42], as adapted by Thomas et al. [43]. This quality assessment tool evaluates representativeness, randomization, comparability, credibility of data collection instruments, attrition rate and attributability to intervention.

## Results

**Figure 1:**
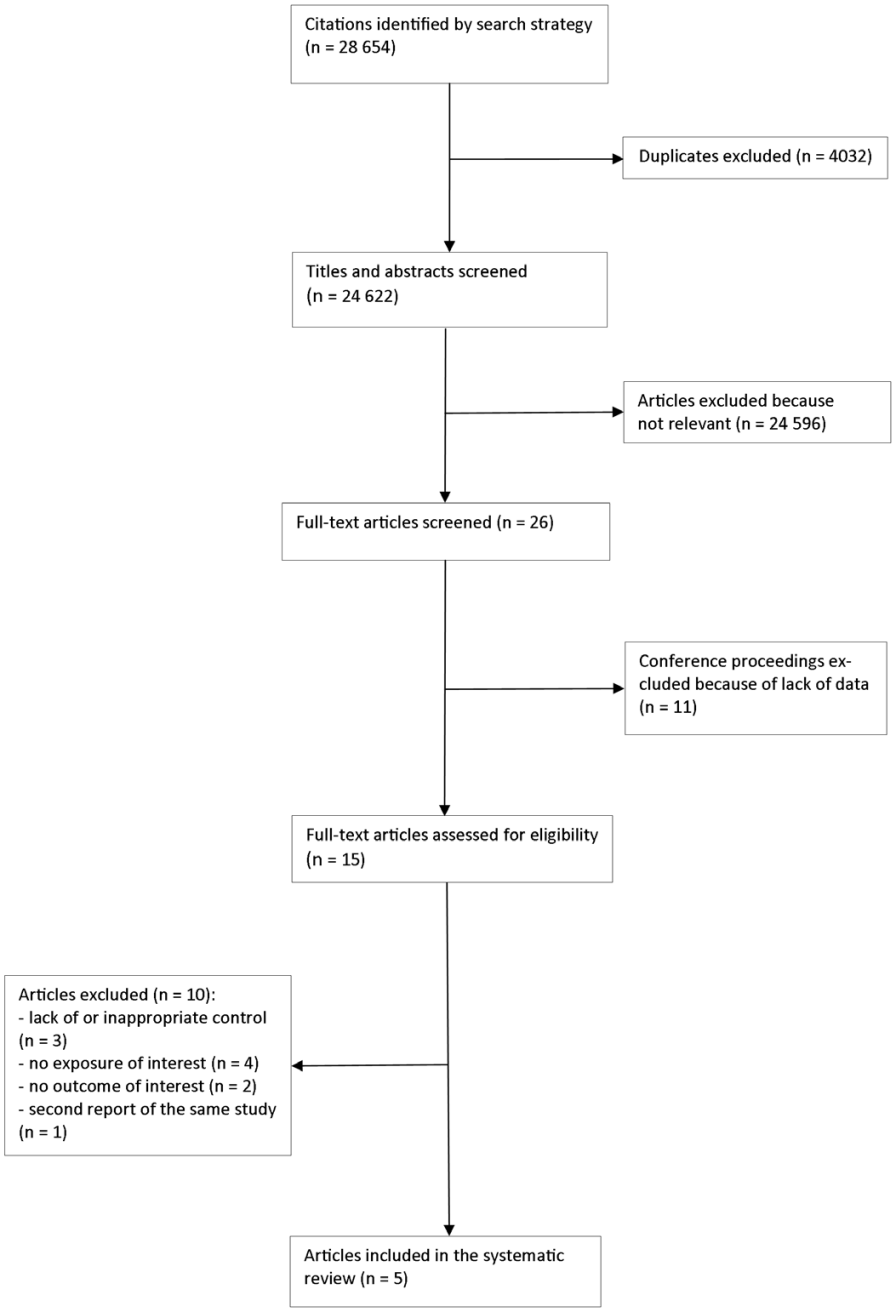
Flow-diagram of the study selection process.

We identified 28 654 citations. Five studies met the inclusion criteria (Table S1) [36], [37], [44]–[46]. One study was reported twice, in the form of a journal article and thesis [36], [37]. The study selection process is presented in the form of an adapted PRISMA flow-diagram (Figure 1). We will first present the description of the included studies, their quality assessment and types of interventions and then the reported outcomes, i.e. the uptake of integrated interventions.

### Description of Included Studies

The studies were conducted in sub-Saharan Africa: a cluster randomized controlled trial and a stepped wedge design evaluation in Zambia [37], [46], a before and after study in Kenya [44], a before and after study in Malawi [45], and a retrospective cohort study in South Africa [47]. All studies were conducted in low-income countries apart from the South-African study (an upper-middle income country).

### Quality Assessment

The suitability of study design (Table S2 and Table S3) was rated as A for one study [37], B for two studies [46], [47], and C for two studies [44], [45].

The included cluster randomized controlled trial had a low risk of bias (Table S2). Of the remaining studies, one study met five [46], one study met four [47], and other two studies met two of the six quality assessment criteria (Table S3) [44], [45].

### Interventions

The studies were conducted in different periods between 2001 and 2008, in urban settings except for one study set in rural Malawi [45]. The included studies evaluated the effectiveness of integration of HIV testing and structured nevirapine assessment at labor ward [37], integration of ART provision [46], [47] or HIV testing and counseling with antenatal care [44], [45].

### Integration of HIV Testing in Antenatal Care

We found one study from Kenya and one from Malawi that provided data before and after the integration of HIV testing within antenatal care [44], [45]. In both studies, opt-in rapid testing was used.

In a Kenyan program, PMTCT interventions were first partially integrated in antenatal care. Women received general information on the PMTCT program in antenatal care [44]. They were then referred for counseling and implementation of the PMTCT steps to a counselor located in a separate room in the same hospital and to a general hospital laboratory for routine antenatal tests including HIV testing. Women identified as HIV positive had to see the counselor again at 34 weeks of gestation to receive nevirapine for both themselves and their infants. Women who came to the labor ward were asked if they took nevirapine and given a new dose, if necessary. All infants born to HIV positive mothers at the hospital received nevirapine. In the following, intervention period, women were tested in a side laboratory in the antenatal care. The same nurse-counselor provided counseling, HIV testing, post-test HIV counseling and routine antenatal care preventive interventions [44].

In the Malawian program, HIV counseling and testing were offered at a voluntary counseling and testing center, located in the outpatient department. Subsequently, HIV testing and counseling were integrated with antenatal care. Nevirapine was provided to women and infants at labor ward before and after the integration [45].

### Integration of HIV Testing and Structured Nevirapine Assessment with Labor Ward Care

The cluster randomized controlled trial included 7664 women delivering in 12 public-sector delivery centers in Lusaka, Zambia. Six clinics were randomly assigned to the intervention arm and six to the control arm. Midwifes implemented the services [37].

At the intervention sites, women of unknown serostatus received opt-in rapid HIV testing at the labour ward and nevirapine tablet (200 mg) if identified as HIV positive. HIV seronegative women were offered a calcium tablet to avoid disclosure of the HIV status and potential stigmatization of HIV positive women. Previously identified HIV positive women were provided with nevirapine in antenatal care and were administered a structured nevirapine adherence assessment at the labor ward. They were informed about the purpose of nevirapine and were asked if they were prepared to receive it at the labor ward. If women did not ingest the prophylaxis and had indicated their willingness to take it at the labor ward, they received nevirapine in the labor ward.

At the control clinics, only previously identified HIV positive women received nevirapine in the labor ward if they did not take the tablet provided in antenatal care. To establish their adherence, midwifes would informally ask if they had taken the nevirapine.

All infants born to HIV positive mothers had to be provided with nevirapine syrup (2 mg/kg) in both the control and interventional clinics before hospital discharge.

The primary outcome of the study was maternal-infant nevirapine coverage. Maternal coverage was determined by the nevirapine testing of the umbilical cord blood specimens collected anonymously. Infant coverage was determined based on the report of nevirapine administration in the patient’s record. The maternal-infant nevirapine coverage was measured in both control and interventional clinics at baseline and intervention period.

### Integration of ART Provision with Antenatal Care

A stepped wedge evaluation from Zambia and a retrospective observational study from South Africa evaluated integration of ART (i.e. highly active antiretroviral therapy (HAART)) provision in antenatal care [46], [47].

The Zambian stepped wedge design study compared the integration of the ART provision in antenatal clinics and referral to local ART clinics [46]. The data was collected from the same eight antenatal clinics before and after the roll-out of integrated care.

HIV positive women with CD4 cell counts less than or equal to 250 cells/µl were eligible for ART. In the pre-intervention period, eight antenatal sites referred ART eligible women attending antenatal care to ART clinics. ART clinics and antenatal care were located at the same healthcare facility. Peer educators provided additional education to ART eligible women and accompanied them to the ART clinic.

In the intervention period, antenatal clinics started providing ART in antenatal care. The intervention was rolled-out gradually with one new site every month. Antenatal care provided ART services one to two days per week. Both control and intervention clinics used the same schedule of visits, laboratory evaluations, record systems, and quality assurance systems. The same cadre of providers worked before and after the intervention: a clinical officer, a nurse, and a peer educator.

The South African retrospective cohort study compared three different models of integrated care [47]. In one facility, a doctor provided ART twice per week (i.e. integrated care). In another facility, women were referred from antenatal care to the HIV clinic located in a different building but in the same healthcare facility (i.e. proximal partially integrated service). In the last two clinics, women were referred to the HIV clinic situated in a different healthcare center not more than 5 km away from the antenatal care clinic (i.e. distal partially integrated service).

Women with CD4 cell counts equal to or less than 200 cells/µl were considered eligible for ART. All antenatal care facilities routinely performed HIV counseling with opt-in testing and prophylaxis for mothers and infants.

### Outcomes

#### HIV testing in antenatal care

The two before and after studies that assessed integration of HIV testing in antenatal care reported significant increases in testing uptake after the integration [44], [45].

In the Kenyan before and after study, significantly more women received information on PMTCT (92% versus 77%, p<0.001), were tested (76% versus 62%, p<0.001) and learned their status (66% versus 55%, p<0.001) when comparing the intervention (n = 4089) and the control group (n = 4142) [44].

In the Malawian before and after study, the proportion of pregnant women who were tested significantly increased from 52.6% before the integration (n = 196) to 78.7% after the integration (n = 1063), (p<0.001) [45].

The full integration of the ART provision in antenatal care did not have a significant impact on the testing uptake in the Zambian stepped wedge study (97.9% versus 98.4%, p = 0.51) [46].

#### Nevirapine coverage

In the Zambian cluster randomized controlled trial, the proportion of mother-infant pairs receiving nevirapine at the control sites declined from 53% in the baseline period to 43% in the intervention period (range of difference in coverage from –13% to 0%). The maternal-infant nevirapine coverage increased from 42% to 52% (range of difference in coverage from –10% to +33%) at the intervention sites. The relative risk of mother-infant nevirapine coverage in the intervention arm compared to the control was 0.89 at baseline and 1.22 at the end of the intervention period. This change resulted in a ratio of relative risks of 1.37 (RR 1.37, bootstrapped 95% CI, 1.04–1.77). According to the subgroup analysis assessing the effect of each of the two implemented interventions, rapid HIV testing at labor ward was associated with an absolute increase in nevirapine coverage of 16% (range 4 to 25%) in the treatment clinics (from 0% at baseline). The structured assessment of nevirapine adherence was associated with a 4% increase in coverage in the treatment clinics (from 63% at baseline to 67% during the intervention period), compared to a 9% drop in coverage in the control clinics (from 74% at baseline to 65% during the intervention period) [37].

Two before and after studies, evaluating impact of integration of HIV testing in antenatal care on nevirapine coverage at labor ward had mixed results. The acceptance of nevirapine at the labor ward did not significantly change after the integration of HIV testing (92% versus 97%, p = 0.4) in the Malawian program [45]. The Kenyan study reported a significant increase in nevirapine uptake (57% versus 70%, p<0.001) [45]. However, in the Malawian study, nevirapine was only dispensed at the labor ward and in the Kenyan program, nevirapine was dispensed in both antenatal care and at the labor ward [44], [45].

#### ART coverage, initiation and duration

In the Zambian stepped wedge study, the proportion of women who were tested for HIV, who underwent the CD4 count measurement and who were eligible for ART did not differ significantly between the control (n = 716) and the intervention (n = 846), (98.4% versus 97.9%, 85% versus 85.1% and 27.6% versus 26.5%, respectively). The complete integration of ART provision in antenatal care was associated with higher proportion of women enrolled in HIV care (44.4% versus 25.3%, p<0.001) and provided with ART (32.9% versus 14.4%, p<0.001) [46].

There was no significant difference between control and intervention either in the median gestational age of ART initiation (27.1 versus 27.7 weeks, p = 0.4) or in mean weeks of ART initiation before delivery (10.8 versus 10.0 weeks, p = 0.3). Ninety days retention in care after the initiation of ART therapy was similar in both groups (87.8% in the intervention and 91.3% in the control, p = 0.3) [46].

In the South African cohort study, the proportion of eligible women initiating ART in integrated (n =  227), proximal (n = 159) and distal (n = 130) care did not differ significantly: 55%, 48% and 47%, respectively (p = 0.29). No significant difference was found between integrated, proximal or distal care either in the median gestational age of initiation of ART (32 weeks (range 28–35) versus 33 weeks (range 29–36) versus 31 weeks (range 26–34), p = 0.11) or the proportion of women who stayed on ART for more than eight weeks compared to those who received eight weeks or less of ART (50% versus 42% versus 52%, p = 0.96). The duration of ART before the delivery was eight weeks in 48%, between four and eight weeks 32% and less than 4 weeks in 20% of women [47].

#### Overall uptake of PMTCT interventions in integrated programs

The five studies evaluated integration of a single perinatal PMTCT intervention and reported four key outcomes: uptake of HIV testing, nevirapine and ART, and ART initiation and ART duration.

In the two studies evaluating integration of HIV testing into antenatal care, the testing coverage was 76% and 79%, respectively [44], [45]. A high-quality study, cluster randomized controlled trial, while successful in achieving a 10% increase in nevirapine coverage fell 30% short of the 80% coverage target [37]. In Zambian study, only 37% of eligible women enrolled into ART care and only 24% started the therapy [46]. In South African integrated PMTCT program, only 55% of eligible women initiated ART, 26% received ARV prophylaxis, and 19% did not receive any antiretroviral intervention before delivery in integrated care [47].

#### Cost-effectiveness, impact on human resources, stigma and quality of care, attendance of services integrated with PMTCT interventions

None of the included studies compared the cost-effectiveness, impact on human resources, stigma, quality of care or attendance of services integrated with PMTCT interventions between the different models of care.

## Discussion

Our review shows there is very limited, non-generalizable evidence of improved PMTCT intervention uptake in integrated PMTCT programs. None of the included studies evaluated integration of the whole PMTCT program. Further, the uptake of integrated PMTCT interventions was mostly low, and did not reach the 80% target set by UNGASS.

We found no information about the barriers to PMTCT uptake among the participants in the included studies, although the women’s preferences are critical in the uptake of the PMTCT [48], [49]. Two included studies [37], [44] indicated related research [50], [51] on reasons for loss to follow up in integrated PMTCT programs which reported wide range of motives ranging from lack of staff, late labor ward presentation to fear of partner violence and stigma. In the included Kenyan study, partners of women participating in integrated care were also invited to counseling and testing, but only 1% of them attended the clinic [44]. Such a low uptake is of great concern given the impact that male involvement could have on pregnant women’s participation in the PMTCT programs [52].

Quality of the included studies varied from a cluster randomized controlled trial with low risk of bias to before and after studies meeting only two out of the six quality criteria. The included studies were heterogeneous, presenting integration of various PMTCT interventions in different settings. Only the Zambian cluster randomized control trial evaluated labor ward-based integrated PMTCT care. In this study, the control group consisted of labor wards without the targeted PMTCT intervention (i.e. HIV testing and structured nevirapine adherence assessment). Other studies mostly used partially integrated care (i.e. referral to PMTCT program) as a control. In most of the studies, women and children in need of PMTCT intervention were referred to separate PMTCT services in the same hospital or on the same premises where antenatal care clinics were located [44]–[46]. A study with PMTCT interventions offered either in a separate building on the same premises as antenatal clinic or within 5-km radius [47], showed no significant difference in intervention uptake in regards to distance of partially integrated PMTCT services. While it is encouraging that distances up to 5 km did not seem to be critical with regards to uptake, both types of service delivery fell short of the UNGASS target and as such do not answer the question of how to organize the services effectively.

Our findings are consistent with the Cochrane review on strategies for integrating primary health services at the point of delivery in developing countries, which was also not able to provide a general message about their effectiveness [19]. Assessing effects of integration of services is not challenging just because of lack of studies but also because of lack of comparability of approaches. One of the key concerns regarding the integration of PMTCT program is its impact on the quality of existing healthcare services [53], [54]. None of the included studies reported this outcome. A recently published systematic review assessed the impact of PMTCT integration on the quality of maternal healthcare services and reported both positive and negative effects and limited evidence base [53].

Several systematic reviews have assessed the effectiveness of integration of different healthcare services. Two systematic reviews assessing the effectiveness of linking family planning and sexual and reproductive health with HIV/AIDS interventions reported positive effects of integration on the uptake of HIV testing [26], [27]. They also stated that the included studies only evaluated integration at service delivery level and were not specifically designed to compare the benefit of providing integrated services with the same services offered separately.

Our systematic review has a number of strengths. We undertook an extensive search of numerous electronic databases without the use of specific search terms for integrated care, methodological filters or language restrictions and searched for relevant indexed peer-reviewed academic and grey literature. We built on high-quality, internationally recognized Cochrane systematic review methodology and expanded the inclusion of study designs with a greater risk of bias (observational studies) but which may provide additional insight into ‘real-world’ effectiveness [55]. The operational research focused on health services and health systems often warrants alternative study designs given the complexity and dynamic nature of these organizational units [56]. The limitations of this review include the low number of studies of variable quality that were highly heterogeneous, which prevented us from reaching a conclusion on the effectiveness of different modes of integrated PMTCT programs. However, given the complex and heterogeneous nature of healthcare delivery and integration, it is debatable if research focused on these topics can in fact be sufficiently homogenous to allow for a uniform, straightforward conclusion about the effectiveness of integrated programs. We are also aware that the definition of integrated healthcare we use, focused mainly on location of service delivery, may have resulted in the omission of other potentially relevant articles.

The question of integration may be seen as outdated since recommendations to integrate PMTCT programs is widely embedded into global and national policies and strategies for fighting the HIV epidemic [18], [57]. Our study, however, reveals a highly problematic position showing that evidence base on the effectiveness of integrated care compared to non- or partially integrated is scarce at best, with no clarity on modality of integration and all models failing to achieve target coverage.

While it is plausible to anticipate beneficial effects of integrated PMTCT programs through mechanisms of improving access and increasing uptake of PMTCT interventions; we agree with the current guidance proposed by international health organizations stating that future integration of PMTCT programs to other services must carefully consider system readiness for integration and the epidemiological context [12], [17], [18]. Outcomes other than intervention coverage such as cost-effectiveness, impact on quality of care, human resources and stigma [48], [58] and the wide range of contexts in which these programs are implemented should also be considered [59]–[62]. The effectiveness of PMTCT program delivery models depends also on HIV prevalence, health seeking behavior and the dominant mode of horizontal HIV transmission among attendees.

Considering the size of the problem, potential for dramatic impact of PMTCT interventions and the corresponding scale of investment into HIV care and prevention, policy makers must ensure urgent prioritization of rigorous research on integration of PMTCT.

## Supporting Information

Table S1 **Characteristics of included studies.**
(DOCX)

Table S2 **Suitability of study design and risk of bias in cluster randomized controlled trial.**
(DOCX)

Table S3 **Suitability of study design and quality assessment.**
(DOCX)
