# Supplementary material for: Integrating Prevention of Mother-to-Child HIV Transmission Programs to Improve Uptake: A Systematic Review
Source: PLoS One. 2012 Apr 27;7(4):e35268. doi: 10.1371/journal.pone.0035268 (PMC3338706; doi:10.1371/journal.pone.0035268)
Supplement: Table S1 — Characteristics of included studies. (DOCX) [file pone.0035268.s001.docx]

| **Author, Year of publication, Country, Study duration** | **Study design** | **Participants and sample size** | **Intervention** | | **Comparison** | **Primary outcomes reported** | **Results** |
| --- | --- | --- | --- | --- | --- | --- | --- |
| Kasenga 2009 [[44](#_ENREF_44)], Malawi, Jan-05–Dec-07 | Before and after | Women in ANC Intervention: 1063 Control: 196 | | HIV testing and counseling & services integrated within ANC; Women received infant feeding counseling; Safe delivery; Women received ARV prophylaxis in LW | **Non-integrated:** HIV testing of women not provided in ANC but in separate VCT unit; Women received infant feeding counseling; Safe delivery; Women received ARV prophylaxis in LW | ANC: 1.Percentage of women tested in ANC; LW: 2.Percentage of women receiving ARV prophylaxis | Control vs intervention: 1.53% vs 79%, p<0.001; 2.92% vs 97%, p=0.40 |
| Killam 2010 [[45](#_ENREF_45)], Zambia, Jul-07–Jul-08 | Stepped wedged | Women in ANC Intervention: 17 619 Control: 13 917 | | HIV testing of women at ANC; Women received ART in ANC | **Partially integrated:** HIV testing of women at ANC; Women referred for ART from ANC to ART clinic | ANC: 1.Percentage of women tested in ANC; 2.Percentage of HIV positive women who had a CD4 test; 3.Percentage of women enrolled in ART clinic; 4.Percentage of women receiving HAART as part of PMTCT; 5.Percentage of women on care at 90 days of follow up | Control vs intervention: 1.98.4% vs 97.9%, p=0.51; 2.85% vs 85.1%, p=0.98; 3.25% vs 44%, p<0.001; 4.14% vs 33%, p<0.001; 5.91% vs 88%, p=0.3 |
| Megazzini 2010 [[36](#_ENREF_36)], Zambia, Oct-05–Jan-06 | cRCT | Women at LW Intervention: 2435 Control: 2106 | | HIV testing of women at ANC; Women received ARV prophylaxis at ANC; HIV testing of women of unknown serostatus in LW; Nevirapine administration as indicated; Formal nevirapine adherence assessment | **Partially integrated:** HIV testing of women at ANC; Women received ARV prophylaxis in ANC; No formal nevirapine adherence assessment | LW: Percentage of women/infants receiving ARV prophylaxis | Baseline period/intervention period: Control: from 53% to 43% (difference ranged -13% to 0%); Intervention: from 42% to 54% (difference ranged -10% to +33%) |
| Stinson 2010 [[46](#_ENREF_46)], RSA, Jan-Dec 2005 | Cohort | Women in ANC Fully integrated one point: 4823; Partially integrated proximal: 4783; Partially integrated distal: 5381 | | HIV counseling, testing and ARV prophylaxis; Women received ART at the ANC, twice per week | **Partially integrated proximal:** HIV counseling, testing and ARV prophylaxis; Women referred for ART to HIV clinic in the same health facility. **Partially integrated dista**l: HIV counseling, testing and ARV prophylaxis; Women referred for ART to HIV clinic in another health facility within 5 km | ANC: 1.Percentage of women tested in ANC; 2.Percentage of HIV positive women who had a CD4 test; 3.Percentage of women starting HAART as part of PMTCT; 4.Percentage of HAART eligible women receiving ARV prophylaxis | Integrated vs proximal vs distal: 1.Total: 88.1%; 2.Total: 97%; 3.55% vs 48% vs 47%, p=0.29; 4.26% vs 28% vs 29% |
| Van’t Hoog 2005 [[43](#_ENREF_43)], Kenya, Nov-01– Aug-03 | Before and after | Women in ANC Intervention: 4089 Control: 4142 | | Nurse counselor provided all PMTCT interventions in ANC; HIV testing of women provided in ANC ; Women received ARV prophylaxis at ANC ; Infant ARV prophylaxis dispensed at ANC; ARV prophylaxis dispensed any time from the 2nd trimester; Women received infant feeding counseling at ANC; Safe delivery in LW; Women received ARV prophylaxis in LW; Infants on ARV prophylaxis in LW | **Partially integrated:** HIV testing of women not provided in ANC but in off-site laboratory; Counseling not provided in ANC but in a separate location by a counselor; ARV prophylaxis for women and infant provided from the counselor at 34 weeks; Women received infant feeding counseling from the counselor; Safe delivery in LW; Women received ARV prophylaxis in LW; Infants on ARV prophylaxis in LW | ANC: 1.Percentage of women who received information on PMTCT; 2.Percentage of women tested; 3.Percentage of women who received their test result LW: Percentage of women receiving ARV prophylaxis | Control vs. intervention: 1.77% vs 92%, p<0.001; 2.62% vs 76%, p<0.001; 3.89% vs 90%, p=0.23; 4.57% vs 70%, p<0.001 |

ANC, antenatal care; ART, antiretroviral therapy; ARV, antiretroviral; cRCT, cluster randomized controlled trial; HAART, highly active antiretroviral therapy; LW, labor ward; PMTCT, prevention of mother to child transmission; RSA, Republic of South Africa; VCT, voluntary counseling and testing; vs, versus.
