# Supplementary material for: Integrating Prevention of Mother-to-Child HIV Transmission Programs to Improve Uptake: A Systematic Review
Source: PLoS One. 2012 Apr 27;7(4):e35268. doi: 10.1371/journal.pone.0035268 (PMC3338706; doi:10.1371/journal.pone.0035268)
Supplement: Table S2 — Suitability of study design and risk of bias in cluster randomized controlled trial. (DOCX) [file pone.0035268.s002.docx]

| **Yes/No/Unclear** | Megazzini 2010 [[36](#_ENREF_36)] |
| --- | --- |
| **Suitability of study design** | Cluster randomized controlled trial |
| Category A: The study design includes concurrent comparison groups AND prospective measurement of exposure and outcome. Category B: Retrospective study design with concurrent comparison group OR the study design includes at least two 'before' measurements and at least two 'after' measurements but no concurrent comparison group. Category C: The study design involves single 'before' and 'after' measurements with no concurrent comparison group. Category D: The study design involves measurements of exposure and outcome made at a single point in time. | A |
| **Risk of bias** |  |
| Free of recruitment bias? | Yes |
| Free of baseline imbalance? | Yes |
| Free of loss of clusters? | Yes |
| Free of incorrect analysis? | Yes |
| Comparability with individually randomized trials? | No |
