# Supplementary material for: Integrating Prevention of Mother-to-Child HIV Transmission Programs to Improve Uptake: A Systematic Review
Source: PLoS One. 2012 Apr 27;7(4):e35268. doi: 10.1371/journal.pone.0035268 (PMC3338706; doi:10.1371/journal.pone.0035268)
Supplement: Table S3 — Suitability of study design and quality assessment. (DOCX) [file pone.0035268.s003.docx]

| **Yes/No/Unclear** | Kassenga 2009 [[44](#_ENREF_44)] | Killam 2010 [[45](#_ENREF_45)] | Stinson 2010 [[46](#_ENREF_46)] | Van’t Hoog 2005 [[43](#_ENREF_43)] |
| --- | --- | --- | --- | --- |
| **Suitability of study design** | Before and after | Stepped-wedge | Retrospective cohort | Before and after |
| Category A: The study design includes concurrent comparison groups AND prospective measurement of exposure and outcome. Category B: Retrospective study design with concurrent comparison group OR the study design includes at least two 'before' measurements and at least two 'after' measurements but no concurrent comparison group. Category C: The study design involves single 'before' and 'after' measurements with no concurrent comparison group. Category D: The study design involves measurements of exposure and outcome made at a single point in time. | C | B | B | C |
| **Methodological quality criteria** |  |  |  |  |
| **Representativeness:** Were the study samples randomly recruited from the study population with a response rate of at least 60% OR were they otherwise shown to be representative of the study population? | YES | YES | YES | YES |
| **Randomization:** Were participants, groups or areas randomly allocated to receive the intervention or control condition? | N/A | NO | N/A | N/A |
| **Comparability:** Were the baseline characteristics of the comparison groups comparable OR if there were important differences in potential confounders were these appropriately adjusted for in the analysis? If there is no comparison group this criterion cannot be met. | UNCLEAR | YES | YES | UNCLEAR |
| **Credibility of data collection instruments:** Were data collection tools shown to be credible, e.g. shown to be valid and reliable in published research, OR in a pilot study, OR taken from a published national survey, OR recognized as an acceptable measure (such as biochemical measures of smoking). | YES | YES | YES | YES |
| **Attrition rate:** Were outcomes studied in a panel of respondents with an attrition rate of less than 30% OR were results based on a cross-sectional design with at least 200 participants included in analysis in each wave? | NO | YES | YES | NO |
| **Attributability to intervention:** Is it reasonably likely that the observed effects were attributable to the intervention under investigation? This criterion cannot be met if there is evidence of contamination of a control group in a controlled study. Equally, in all types of study, if there is evidence of a concurrent intervention that could also have explained the observed effects and was not adjusted for in analysis, this criterion cannot be met. | UNCLEAR | YES | NO | UNCLEAR |
